# The Insufficiency of Norm-Referenced Writing Assessment for Identifying Writing Weaknesses in Children Who Are Deaf and Hard of Hearing

**DOI:** 10.1044/2025_LSHSS-25-00009

**Published:** 2025-07-25

**Authors:** Brittany Grey, Marren C. Brooks, Emily A. Lund, Krystal L. Werfel

**Affiliations:** a Center for Childhood Deafness, Language, and Learning, Boys Town National Research Hospital, NE; b Department of Communication Sciences and Disorders, University of South Carolina, Columbia; c Davies School of Communication Sciences and Disorders, Texas Christian University, Fort Worth

## Abstract

**Purpose:**

This study examined the internal consistency reliability, interrater reliability, and concurrent validity of the norm-referenced Test of Early Written Language–Third Edition (TEWL-3) to determine if it is an appropriate measure to use when determining if elementary children who are deaf and hard of hearing (DHH) meet grade-level writing expectations.

**Method:**

Participants included 111 second-grade children across three groups: children with typical hearing (CTH), children who use cochlear implants, and children who use hearing aids. Reliability and validity were compared across all groups.

**Results:**

Reliability was very strong across groups. CTH followed expected patterns of validity, whereas patterns of validity differed for DHH. Results indicated, on average, that all groups performed in the average or above average ranges on the TEWL-3 but in the not proficient range on the 6 + 1 Trait Writing Rubric: Grades K–2.

**Conclusions:**

Given the purpose of norm-referenced and criterion-referenced measures and the differences in performance on the TEWL-3 and the 6 + 1 Trait Writing Rubric: Grades K–2 across groups, speech-language pathologists should not rely on norm-referenced writing assessments to make eligibility decisions for specialized writing intervention services, especially for the DHH population.

**Supplemental Material:**

https://doi.org/10.23641/asha.29618573

As children advance in their education, writing skills become the backbone of academic success. However, the National Assessment of Educational Progress (NAEP) data report that students demonstrate poor writing abilities throughout their academic years. In 2011, only 32% of fourth-grade students' (White et al., 2015) and 24% of eighth- and 12th-grade students' writing skills were considered proficient (National Center for Education Statistics [NCES], 2012). For children who have diagnoses that are known to impact language, such as those who are deaf and hard of hearing (DHH), it is even more important to monitor writing skills. The purpose of this study was to evaluate the reliability and validity of a commonly used, norm-referenced writing measure for children who were DHH.

## Reading Outcomes for Children Who Are DHH

Although reading outcomes for children who are DHH have improved over the past several decades, those outcomes are not equivalent to outcomes for children with typical hearing (CTH) matched for important variables like socioeconomic status and maternal education. Word-level reading performance is often measured via norm-referenced tests, and many researchers report that children who are DHH, utilize amplification, and use spoken English perform within 1 *SD* of the mean for the normative test sample (Antia et al., 2020; Mayer et al., 2016; Spencer & Tomblin, 2009; Werfel & Hendricks, 2023). However, when studies employ a comparison group of CTH (usually matched for variables beyond age like maternal education and geographic location), children who are DHH often have a significantly lower performance than their counterparts with typical hearing. Studies conducted with school-aged children 10–15 years prior to the writing of this article report that the mean single-word reading performance of children with cochlear implants (CIs) was within at least 1.25 *SD*s of normative sample means on norm-referenced assessments (Geers & Hayes, 2011; Johnson & Goswami, 2010; Spencer & Tomblin, 2009). However, in a study that included an age-matched group with typical hearing rather than relying on test norms, Nittrouer et al. (2012) found that kindergarten children with CIs performed significantly lower than their typical hearing peers. In more recent studies, similar patterns are observed. Mayer et al. (2016) found that 55% of children with CIs performed within the range expected for children of the same age, according to the test, on single-word reading. Antia et al. (2020) determined children with CIs and hearing aids (HAs) who used spoken English performed in the range of normal according to test norms in kindergarten through second grade on word reading tasks. When Werfel and Hendricks (2023) compared the performance of children with CIs to a control group of CTH matched for age, nonverbal intelligence, and maternal education, they found a significant difference between groups on single-word reading. Thus, even when children who are DHH perform within the average range on reading measures, that finding does not indicate that they are performing at the same level as their peers with typical hearing who are matched for other important variables like maternal education (as a proxy for socioeconomic status), even in light of recent technological advances.

Children who are DHH demonstrate a similar pattern of performance on reading comprehension measures as they do on word reading tasks. Previous studies over the past 20 years determined that school-aged children who were DHH, utilized amplification, and used spoken English often performed within the average range on norm-referenced reading comprehension measures according to testing norms (Antia et al., 2020; Bharadwaj & Barlow, 2020; Geers & Hayes, 2011; Mayer & Trezek, 2018, 2024; Mayer et al., 2016; Spencer et al., 2003; Tomblin et al., 2020; Walker et al., 2020). However, many researchers reported that the scores of DHH fell on the lower end of average (Antia et al., 2020; Geers & Hayes, 2011; Johnson & Goswami, 2010; Mayer & Trezek, 2024; Spencer et al., 2003). In a review of 14 studies that utilized standard scores from norm-referenced assessments, Mayer and Trezek (2018) reported that most CI groups from ages 5 through 18 years performed within the expected range for children of the same age on reading comprehension tasks when compared to the normative sample. More recently, Bharadwaj and Barlow (2020) found that third- through fifth-grade students who were DHH and used spoken English demonstrated average reading comprehension outcomes on norm-referenced assessments, with no significant differences in performance between CI users and HA users. Additionally, Antia et al. (2020) reported that kindergarten children who were DHH and used spoken English yielded reading comprehension scores similar to the normative sample. However, in the latter study, the spoken English DHH group's reading comprehension outcomes decreased to low average in first grade and below average in second grade according to test norms (Antia et al., 2020). Furthermore, Mayer and Trezek (2024) reported CI users in fourth through 12th grades yielded mean standard scores in the low average range according to assessment norms from norm-referenced reading comprehension measures.

When employing a control group of age-matched peers, Spencer et al. (2003) reported that children with CIs demonstrated reading comprehension skills within the average range according to testing norms, but mean scores were well below those from the control group. Johnson and Goswami (2010) compared the reading comprehension skills of CI users and HA users to reading age–matched CTH. They reported that only the early implanted (i.e., before 39 months) CI users yielded standard scores within the average range according to testing norms, which was still 1 *SD* below the reading age–matched typical hearing group (Johnson & Goswami, 2010). On the contrary, no group differences were reported between the reading comprehension skills of HA users with mild-to-moderate hearing loss and a control group of age-matched typically hearing peers (Tomblin et al., 2020; Walker et al., 2020).

Whereas researchers from aforementioned studies measured reading comprehension skills with norm-referenced assessments, Nittrouer et al. (2012) utilized a criterion-referenced measure and employed an age-matched control group of CTH. They found that typical hearing kindergarten students outperformed both kindergarten HA users and CI users, with CI users demonstrating significantly poorer reading comprehension outcomes than typical hearing peers (Nittrouer et al., 2012). Over the past 20 years, researchers determined that most children who were DHH and used spoken English performed close to or within the average range on reading comprehension outcomes according to normative samples on norm-referenced measures. However, when the reading outcomes for DHH and CTH groups (that were matched for variables such as age, nonverbal intelligence, and maternal education) were compared, a significant performance gap existed.

## Writing Outcomes for Children Who Are DHH

When reporting on the literacy skills of children who are DHH, most research highlights reading results as opposed to writing results. However, as children advance in their academic years, assignments often require writing, which begin with spelling. When studies used norm-referenced spelling assessments, most researchers reported that children who utilized CIs or HAs and used spoken English achieved standard scores within the average range according to testing norms (Spencer et al., 2004; Tomblin et al., 2020; Werfel & Hendricks, 2023). When Tomblin et al. (2020) examined spelling outcomes from a norm-referenced measure between HA users with mild to moderately severe hearing loss and a control group of age-matched CTH, they reported no group differences. Additionally, Spencer et al. (2004) reported that high school CI users' average spelling standard scores were in the mid- to high-average range according to testing norms. However, when researchers employed a comparison group of age-matched typical hearing peers, CI users demonstrated poorer spelling outcomes. Werfel and Hendricks (2023) reported that children who used CIs and spoken English performed in the low- to mid-average range on norm-referenced spelling measures, which was significantly below the age-matched comparison group. Group differences in spelling skills were also reported when criterion-referenced assessments were utilized. When children spelled words from picture stimuli, high schoolers with CIs demonstrated significantly poorer spelling accuracy than the age-matched typical hearing control group (Geers & Hayes, 2011). Thus, when DHH spelling skills are measured with testing norms from a norm-referenced spelling assessment, they tend to perform within the range expected for their age. However, CI users' average performance on norm-referenced measures does not translate to commensurate performance with typical hearing counterparts, despite groups being matched for variables beyond age.

Albeit sparse, reported writing outcomes for school-aged children who were DHH appeared to vary based on the type of assessment used. When writing ability was evaluated with a norm-referenced assessment, children who were DHH tended to score within the average or above-average ranges, whereas when evaluated with a criterion-referenced assessment, significant differences between children who were DHH and their peers emerged. Researchers who utilized norm-referenced assessments to measure the writing skills of children who were DHH and used spoken English reported no group differences between HA users and a control group of typical hearing peers (Tomblin et al., 2020), above-average performance for CI users (Spencer et al., 2004), and average to above-average outcomes for HA and CI participants (Mayer & Trezek, 2023) when compared to the normative sample. On the contrary, when written products were evaluated with a criterion-referenced assessment that included predetermined benchmarks, fewer than half of children who utilized CIs met curriculum-level writing expectations (Mayer et al., 2016). Furthermore, when an age-matched comparison group of CTH was employed, children who utilized CIs and used spoken English demonstrated significantly poorer expository writing skills, wrote fewer words, used fewer morphosyntactic features, and had more difficulty formulating grammatically sound sentences in their written narratives than their typical hearing counterparts (Breland et al., 2022; Geers & Hayes, 2011; Spencer et al., 2003). Given the limited number of studies that examined the writing skills of children who were DHH and used spoken English, more evidence is needed to determine how the type of writing assessment impacts conclusions about the child's skills.

## Measuring Writing Outcomes

The Common Core State Standards (CCSS) in the United States are grade-specific academic goals in mathematics and English language arts that outline the skills children should achieve prior to being promoted to the next grade level. Adopted by 41 states, the CCSS were created to unify state academic standards and to increase the nation's overall academic success from kindergarten through 12th grade (Common Core State Standards Initiative [CCSSI], https://www.thecorestandards.org). According to CCSS for writing in kindergarten through fifth grade, children are expected to increase their use and sophistication of vocabulary, syntax, and development/organization of ideas as they progress through their elementary years. By the end of second grade, the CCSS state that students should have a general idea of text organization. They should be able to compose various types of texts (e.g., opinion, informative/explanatory, narratives) where they introduce the topic, provide details/facts/reasons/thoughts/feelings to support the topic, and state a conclusion. Linking words (e.g., “and,” “because”) and temporal vocabulary (e.g., “first,” “last”) should be included in their compositions. Second-grade students should be utilizing various word forms including collective nouns (e.g., “group”), irregular plural nouns (e.g., “feet”), reflexive pronouns (e.g., “himself”), common irregular verbs (e.g., “sat”), adjectives (e.g., “little”), and adverbs (e.g., “slowly”). Furthermore, children should be able to form, expand, and rearrange both simple and compound sentences. With guidance, second-grade students should be able to edit and revise written work while adhering to capitalization (e.g., names), punctuation (e.g., commas in greetings/closings), and spelling (i.e., use learned spelling patterns) conventions. Additionally, second-grade students should be utilizing multiple sources to gather information when participating in shared research, completing writing projects, and answering questions (CCSSI, https://www.thecorestandards.org). Thus, when making eligibility decisions for specialized writing intervention, speech-language pathologists (SLPs) in the United States should refer to the child's grade specific writing and language CCSS when evaluating performance.

### Norm-Referenced Versus Criterion-Referenced

Two types of assessments are commonly used for measuring writing performance: norm-referenced and criterion-referenced. The purpose of norm-referenced assessments is to compare an individual's performance to the performance of their peers. Norm-referenced assessments are developed by using statistics to determine item selection and identify items that best separate high- versus low-performing children, rather than identify items that represent a child's mastery of a particular skill set (Miller, 1989/2019). The Test of Early Written Language–Third Edition (TEWL-3; Hresko et al., 2012) is a commonly used norm-referenced assessment that measures writing skills for children ages 4–11 years and for those in first through sixth grades.

The purpose of criterion-referenced assessments is to examine an individual's performance on a particular skill against predetermined benchmarks or competencies. Criterion-referenced tests are developed using a hierarchy of behavioral skills or instructional goals, and the content covered is typically more specific than the skills sampled on a norm-referenced assessment (Miller, 1989/2019). The 6 + 1 Trait Writing Rubric: Grades K–2 (Education Northwest, 2021) is a commonly used criterion-referenced assessment that measures writing skills for children in kindergarten through second grade. The 6 + 1 Trait Writing Rubric: Grades K–2 incorporates the traits of writing developed by Culham (2011) and the domains from the 2011 NAEP Writing Assessment Framework and (Bridges, n.d.). Additionally, the 6 + 1 Trait Writing Rubric: Grades K–2 (Education Northwest, 2021) aligns with most of the writing and language CCSS for second grade through these domains: Ideas (i.e., stating a clear idea and providing supporting details), Organization (i.e., including a beginning, middle, and end that work together), Voice (e.g., expressing thoughts and feelings about a topic), Word Choice (e.g., using adjectives and adverbs to create an image), Sentence Fluency (i.e., formulating varied simple sentences and using transition words to aid in flow), and Conventions (i.e., adhering to capitalization, punctuation, and spelling rules/patterns). Therefore, both norm-referenced and criterion-referenced assessments provide valuable information about students' writing abilities.

### Psychometric Properties of Tests

When selecting measures, reliability and validity are two important psychometric criteria to consider. Reliability addresses the degree by which a measure's results are consistent (Fitzner, 2007). Two types of reliability that are important when addressing the reliability of a norm-referenced writing assessment are internal consistency reliability and interrater reliability. Internal consistency reliability measures consistency of results across multiple test items within a selected assessment (Fitzner, 2007) and is commonly measured via Cronbach's alpha. The closer the resulting coefficient is to 1.0, the stronger the internal consistency reliability. Interrater reliability measures how similarly multiple raters assign scores to item responses on a selected assessment (Plante & Vance, 1994) and is commonly measured via Cohen's kappa. The closer the resulting coefficient is to 1.0, the stronger the interrater reliability. When internal consistency reliability and interrater reliability are considered strong, the selected assessment is a reliable option to use with the target population when determining if a child is meeting specific standards.

Validity addresses if a selected assessment accurately measures what it proposes to measure (Plante & Vance, 1994). Specifically, concurrent validity is valuable because it describes the degree by which a selected assessment's results are correlated to results derived from a different assessment at the same point in time (Fitzner, 2007). To show strong concurrent validity, it is helpful to show that a given measure is strongly related to other assessments that purport to measure the same construct, somewhat related to other assessments that measure a related but different construct, and minimally to not related to assessments that measure a construct that is not related. For example, for writing, another writing measure could be used to measure the same construct, a reading measure for a related but different construct, and a mathematics measure for a construct that should be not or minimally related to the target. Concurrent validity is commonly measured via Pearson's correlation. If the selected writing measure has a strong positive correlation with another writing measure (i.e., resulting coefficient close to 1.0), a moderate correlation with a reading measure (i.e., resulting coefficient close to .5), and a weak correlation with a math computation measure (i.e., resulting coefficient below .4), one could conclude that the selected writing assessment accurately measures its intended construct and is a valid option to use with the target population when determining if a child is meeting purposed writing expectations. When selecting assessments to determine if a child is meeting grade-level writing expectations, it is important to consider reliability and validity data of a given measure, which is often reported in testing manuals.

Current NAEP writing data indicate that most school-aged children in the United States, including those who are DHH, do not meet grade-level writing expectations (NCES, 2012; White et al., 2015). Prior research that examined writing outcomes for school-aged children who were DHH and used spoken English demonstrated that children who used CIs have poorer writing outcomes than their typical hearing counterparts on criterion-referenced measures (Breland et al., 2022; Geers & Hayes, 2011; Spencer et al., 2003). However, when the writing outcomes of children who were DHH and used spoken English were evaluated with norm-referenced measures, CI users and HA users performed within the range expected for their age according to testing norms and/or were commensurate with typical hearing peers (Mayer & Trezek, 2023; Spencer et al., 2004; Tomblin et al., 2020). Later academic achievements are often dependent on written assessments and tasks, yet research demonstrates that school-aged children who are DHH and those with typical hearing are not meeting writing proficiency standards in the United States. Therefore, it is important that SLPs address writing skills to the same extent as other areas of language, especially in children who are DHH. The purpose of this study was to evaluate the reliability and validity of a commonly used norm-referenced writing measure with children who were DHH as in CTH and to determine if this norm-referenced writing measure accurately identified children who were DHH who did not meet grade-level writing expectations according to a commonly used criterion-referenced measure.

## Research Questions

1. Does the TEWL-3 have strong internal consistency reliability and interrater reliability for assessing writing in children who are DHH, as in CTH?
2. Does the TEWL-3 have strong concurrent validity for assessing writing in children who are DHH, as in CTH?
3. Do the standard scores derived from the TEWL-3 accurately identify children who are DHH who do not meet grade-level writing expectations according to a criterion-referenced measure and do those outcomes differ from CTH?

## Method

All study procedures were approved by the institutional review board (IRB) of the University of South Carolina, which served as the IRB of record (Pro00077931), with agreement from Texas Christian University and Boys Town National Research Hospital. Prior to participation, guardians provided informed consent. Children provided assent at the start of each session.

### Participants

Participants were part of the Early Language and Literacy Acquisition in Children With Hearing Loss (ELLA) longitudinal study (Lund & Werfel, 2022; Werfel et al., 2023). Inclusion criteria included completion of second grade, use of spoken English as the primary language, nonverbal intelligence quotient (IQ) within the average range, and no additional diagnoses that affect language or cognition (e.g., autism, developmental delay). Additionally, DHH participants had bilateral hearing loss and used amplification with CIs and/or HAs. Information about audiological, educational, and medical diagnoses was gathered from the guardian at the time of testing. Audiograms confirmed the degree of hearing loss for each DHH participant.

The 111 participants for the current study were divided into three groups: CTH (*n* = 50, *M*_age_ = 8.2 years), children who use CIs (*n* = 32, *M*_age_ = 8.3 years), and children who use HAs (*n* = 29, *M*_age_ = 8.5 years). If a child only had one CI, or one CI and one HA, they were categorized as part of the CI group. There was not a statistically significant difference between the groups regarding age in months, mothers' years of education, gender, and race. See Table 1 for more details. Audiological information about the DHH participants is outlined in Table 2.

**Table 1.** Participant demographics by group (means and standard deviations).

Variable	Group			
	CTH (*n* = 50)	CI (*n* = 32)	HA (*n* = 29)	*p*
Age	98.38 (3.33) months	99.28 (3.30) months	101.62 (4.58) months	.008
Biological sex	Boys: 19	Boys: 14	Boys: 16	.333
	Girls: 31	Girls: 18	Girls: 13	
Race and ethnicity	White: 44	White: 30	White: 23	.291
	Black: 2	Black: 1	Black: 3	
	Asian: 1	Asian: 1	Native Hawaiian or Other Pacific Islander: 1	
	Hispanic/Latino: 4	Hispanic/Latino: 3	Hispanic/Latino: 5	
	Prefer not to respond: 3	Prefer not to respond: 1	Prefer not to respond: 4	
Caregiver education level	17.85 (2.32) years	16.62 (2.21) years	16.24 (3.21) years	.023

*Note.* CTH = children with typical hearing; CI = cochlear implant users; HA = hearing aid users.

**Table 2.** Audiological information for the deaf and hard of hearing group.

Variable	Group						
	CI (*n* = 32)			HA (*n* = 29)			*p*
	*M*	*Mdn*	*SD*	*M*	*Mdn*	*SD*	
Age of identification	3.94	1	8.15	15.73	6	18.63	*.073*
Age of first hearing aid	7.65	3	9.47	19.06	11	18.11	*.013*
Age of first implant	24.50	14.50	18.21	–	–	–	–
Age of second implant	27.58	17	20.80	–	–	–	–

*Note.* All ages are represented in months. CI = cochlear implant users; HA = hearing aid users.

### Measures

The purpose of the current study was to evaluate the reliability and validity of the TEWL-3 (Hresko et al., 2012), a norm-referenced measure commonly used to determine eligibility for specialized writing intervention. To assess the concurrent validity of the norm-referenced TEWL-3, the same written product derived from the TEWL-3 Contextual Writing subtest was evaluated using the criterion-referenced 6 + 1 Trait Writing Rubric: Grades K–2 (Education Northwest, 2021). The TEWL-3 Contextual Writing subtest and the 6 + 1 Trait Writing Rubric: Grades K–2 both measure the same construct (written expression). Additionally, concurrent validity was evaluated using another writing measure, the Writing Curriculum-Based Measure (CBM; Hosp et al., 2007). The Writing CBM was selected because it measures a similar construct (writing fluency) but is scored from a separate written product. To evaluate concurrent validity of a separate but highly related construct (reading), we used the Woodcock Reading Mastery Tests–Third Edition (WRMT-III; Woodcock, 2011) Total Reading index. When evaluating concurrent validity, it is also important to consider constructs that should be minimally related to the target construct. In this case, we measured math computation using the Kaufman Test of Educational Achievement–Third Edition (KTEA-3; Kaufman & Kaufman, 2015) Math Computation subtest.

*TEWL-3.* The TEWL-3 (Hresko et al., 2012) is a commonly used norm-referenced assessment that measures writing skills for children ages 4–11 years and for those in first through sixth grades. Participants completed the Contextual Writing subtest of the TEWL-3. All participants completed Form B in second grade. In the TEWL-3 Contextual Writing subtest, the child is tasked with writing a narrative story using a picture prompt. The writing sample is scored on various written elements including theme, characters, organization, vocabulary, dialogue, sentence structure, conventions, and spelling. Each of the 20 items has a 4-point scoring system (i.e., 0–3) with specific criteria for what is required to receive that score. The raw score is the number of total points across 20 items (maximum 60 points). Raw scores were converted to standard scores according to the published test manual (Hresko et al., 2012). Double scoring and reliability for the TEWL-3 Contextual Writing subtest are described below.

*6 + 1 Trait Writing Rubric: Grades K–2.* The 6 + 1 Trait Writing Rubric: Grades K–2 (Education Northwest, 2021) is a criterion-referenced rubric that measures writing skills for children in kindergarten through second grade. Writing categories on the 6 + 1 Trait Writing Rubric: Grades K–2 include Ideas, Organization, Voice, Word Choice, Sentence Fluency, Conventions, and Presentation. There are 23 items, including two optional items, on the 6 + 1 Trait Writing Rubric: Grades K–2. Items are evaluated with a 6-point hierarchy ranging from 1 (*beginning*) to 6 (*exceptional*), with scores of 1 through 3 considered not proficient and scores of 4 through 6 considered proficient. An explanation of each writing trait/item and score is provided on the rubric (Education Northwest, 2021). The total scores from 22 items from the 6 + 1 Trait Writing Rubric: Grades K–2 were used to assess the concurrent validity of the TEWL-3 Contextual Writing subtest. The optional drawing item was not included in the scoring because drawing is not part of the TEWL-3 Contextual Writing protocol.

In addition to assessing the concurrent validity of the TEWL-3 Contextual Writing subtest, the 6 + 1 Trait Writing Rubric: Grades K–2 was also used to determine if standard scores derived from the TEWL-3 Contextual Writing subtest identified DHH participants who did not meet grade-level writing expectations to the same extent as the 6 + 1 Trait Writing Rubric: Grades K–2 did. The first author, a certified SLP, independently scored the same 111 writing samples from the TEWL-3 Contextual Writing subtest using the 6 + 1 Trait Writing Rubric: Grades K–2. The second author, a certified SLP, independently scored one third of each group's (i.e., CTH, CI, and HA) TEWL-3 Contextual Writing subtest writing samples using the 6 + 1 Trait Writing Rubric: Grades K–2. Overall, interrater reliability for the 6 + 1 Trait Writing Rubric: Grades K–2 was high (κ = .903, 95% confidence interval [.840, .965], *p* < .001).

*Writing CBM.* The Writing CBM (Hosp et al., 2007) is a timed 3-min sample written to a prompt. We used the prompt “Today I woke up and … ” The writing sample is scored on the total words written (TWW), words spelled correctly (WSC), and correct writing sequences (CWS). TWW is the number of words written, regardless of spelling or context. TWW includes any letter or group of letters that have a space before or after them. WSC is the number of words spelled correctly, regardless of context. Words are counted as spelled correctly if they are found within the English language, even if they are not correct within the context of the sentence. CWS are two adjacent and correctly spelled words that are acceptable within the context of the written phrase to a native speaker of English. CWS considers punctuation, syntax, semantics, spelling, and capitalization (Hosp et al., 2007). The relation between the TEWL-3 Contextual Writing subtest raw scores and the Writing CBM scores were used to determine the concurrent validity between two assessments that measured similar constructs. Double scoring for the Writing CBM is described below.

*WRMT-III.* The WRMT-III (Woodcock, 2011) measures reading readiness and achievement. Participants completed the Word Identification, Word Attack, Word Comprehension, Passage Comprehension, and Oral Reading Fluency subtests to obtain the Total Reading index score. The relation between the TEWL-3 Contextual Writing subtest raw scores and the WRMT-III Total Reading scores were used to determine the concurrent validity between two assessments that measured separate but related constructs. Double scoring for the WRMT-III Total Reading index is described below.

*KTEA-3.* The KTEA-3 (Kaufman & Kaufman, 2015) measures academic skills in the areas of mathematics, reading, and writing. Participants completed the Math Computation subtest to obtain a standard score. The relation between the TEWL-3 Contextual Writing subtest raw scores and the KTEA-3 Math Computation subtest standard scores were used to determine the concurrent validity between two assessments that measured minimally related constructs. Double scoring for the KTEA-3 Math Computation subtest is described below.

### Procedure

The TEWL-3 Contextual Writing subtest, Writing CBM, WRMT-III Total Reading index, and KTEA-3 Math Computation subtest were administered as part of a larger battery of assessments from the ELLA study protocol. Examiners were certified SLPs who were trained on the administration of each assessment prior to testing. All assessments were given one-on-one in individual testing rooms. After testing sessions completed, assessments were scored by two trained lab members. First, a research assistant with at least a bachelor's degree independently scored the Writing CBM, WRMT-III Total Reading index, and KTEA-3 Math Computation subtest. Then, an SLP double-checked the scoring. On the rare occasion that a disagreement occurred, the lab members discussed it until consensus was reached.

Scoring procedures for the TEWL-3 Contextual Writing subtest differed. Because some of the TEWL-3 Contextual Writing subtest items can be interpreted differently, the first and second authors went through an extensive scoring training prior to scoring written products. In addition to using the TEWL-3 Contextual Writing Expanded Scoring Guide (Hresko et al., 2012), the first and second authors, with direction from the fourth author, established specific scoring guidelines to help with ambiguity. After the scoring training, the first author independently scored all stories from the TEWL-3 Contextual Writing subtest and the second author independently scored a random one third of the TEWL-3 Contextual Writing written products. Our first research question was about reliability of scoring; therefore, the reliability is presented in the Results section below. Writing samples were deidentified in terms of participant characteristics.

When examining results derived from the TEWL-3 Contextual Writing subtest, the authors observed that most standard scores were in the high-average to above-average ranges, yet standard scores did not appear to align with the quality of the written products. Therefore, at least 6 months after scoring the TEWL-3 Contextual Writing stories, the first author scored the same deidentified samples using the 6 + 1 Trait Writing Rubric: Grades K–2 and the second author independently scored a random one third of the written products using the 6 + 1 Trait Writing Rubric: Grades K–2. Categorical descriptors stated in the TEWL-3 manual and on the 6 + 1 Trait Writing Rubric: Grade K–2 were compared.

A third certified SLP independently scored a random one third of the TEWL-3 Contextual Writing samples from each group, without completing the training process and using only the test manual. This step was completed to report reliability that may be expected in typical practice, rather than after extensive training in a research lab. Before completing each analysis, statistical assumptions were checked. If the data did not meet the required assumptions to complete parametric statistical analyses, nonparametric statistical tests were used instead.

### Analysis

For all reliability and validity analyses, results are reported for all participants and broken down by CTH, CI, and HA groups. Two different analyses were completed to address the first research question regarding reliability of the TEWL-3 Contextual Writing subtest. Cronbach's alpha was used to assess internal consistency reliability and Cohen's weighted kappa was utilized to assess interrater reliability. Quadratic weights were used for Cohen's weighted kappa because this analysis allowed for more minor disagreements between raters to receive a milder penalty and for more considerable disagreements to receive a harsher penalty.

The aim of the second research question was to address the concurrent validity of the TEWL-3 Contextual Writing subtest. First, Pearson's correlation was used to assess concurrent validity with the 6 + 1 Trait Writing Rubric: Grades K–2. Preliminary analyses showed that the relation between the TEWL-3 Contextual Writing subtest and 6 + 1 Trait Writing Rubric: Grades K–2 scores is linear, with both variables normally distributed, as assessed by Shapiro–Wilk's test (*p* > .05), and there were no outliers. Second, we initially planned to complete a Pearson's correlation between the TEWL-3 Contextual Writing subtest and the variables of the Writing CBM: TWW, WSC, and CWS. However, the variables from the Writing CBM were not normally distributed (Shapiro–Wilk significant level is less than .05) and, thus, did not meet the required assumptions for Pearson's correlation. Therefore, we used a nonparametric Spearman's rank-order correlation to account for the violation of normality. Visual inspection of the scatterplot showed the relation between variables to be monotonic. Third, Spearman's rank-order correlation was used to assess the relation between the TEWL-3 Contextual Writing subtest and the WRMT-III Total Reading index. Visual inspection of the scatterplot showed the relation between variables to be monotonic. Again, a nonparametric statistical test was used as the WRMT-III Total Reading index scores violated a test of normality (Shapiro–Wilk significant level is less than .05). Finally, a Pearson's correlation was used to assess the relation between the TEWL-3 Contextual Writing subtest and the Math Computation subtest of the KTEA-3. The relation between the variables was linear, there were no outliers, and both variables were normally distributed, as assessed by Shapiro–Wilk's test (*p* > .05).

Descriptive analysis was used to address the third research question regarding if the TEWL-3 standard scores accurately identified children who were DHH who did not meet grade-level writing expectations on a criterion-referenced measure. The number and percentage of children in each group who performed below expectations on the TEWL-3 Contextual Writing subtest and the 6 + 1 Trait Writing Rubric: Grades K–2 score were compared. For the TEWL-3, score interpretations from the manual (e.g., a standard score of 85–115 is considered average) were used. For the 6 + 1 Trait Writing Rubric: Grades K–2, descriptors outlined on the rubric (e.g., a score of 4–6 is considered proficient) were used. Results for children who were DHH were compared to the same analyses with the CTH group.

## Results

Participant results from the TEWL-3 Contextual Writing subtest, 6 + 1 Trait Writing Rubric: Grades K–2, Writing CBM components, WRMT-III Total Reading index, and KTEA-3 Math Computation subtest are detailed by group in Table 3. On the norm-referenced measures, all groups had a mean score within the average or low average range.

**Table 3.** Assessment measures by group.

Assessment	Group						
	CTH		CI		HA		*p*
	*M*	*SD*	*M*	*SD*	*M*	*SD*	
TEWL-3 Contextual Writing	(*n* = 50)		(*n* = 32)		(*n* = 29)		
Raw score	35.56	9.92	34.94	11.05	29.79	10.16	.049
Standard score	116.58	14.86	115.47	16.88	106.83	15.91	.025
6 + 1 Trait Writing Rubric: K–2	(*n* = 50)		(*n* = 32)		(*n* = 29)		
Total score	71.98	14.93	65.72	14.77	60.55	11.19	.002
Average item score	3.27	0.68	2.99	0.67	2.75	0.51	.002
Writing CBM components	(*n* = 49)		(*n* = 32)		(*n* = 29)		
Total words written	23.37	10.24	25.66	12.27	19.69	11.47	.060
Words spelled correctly	19.80	9.73	23.00	12.15	16.62	11.69	.034
Correct word sequences	15.51	10.65	14.97	10.98	10.00	9.80	.026
WRMT-III	(*n* = 49)		(*n* = 32)		(*n* = 28)		
Total reading	110.63	15.79	103.34	18.28	106.39	15.49	.130
KTEA-3	(*n* = 46)		(*n* = 30)		(*n* = 24)		
Math computation	100.76	8.16	95.23	15.20	98.46	13.17	.145

*Note.* CTH = children with typical hearing; CI = cochlear implant users; HA = hearing aid users; TEWL-3 = Test of Early Written Language–Third Edition; CBM = Curriculum-Based Measure; WRMT-III = Woodcock Reading Mastery Tests–Third Edition; KTEA-3 = Kaufman Test of Educational Achievement–Third Edition.

### Research Question 1: Does the TEWL-3 Have Strong Internal Consistency Reliability and Interrater Reliability for Assessing Writing in Children Who Are DHH, as in CTH?

The purpose of our first research question was to determine if the TEWL-3 Contextual Writing subtest is a reliable measure for assessing the writing skills of children who are DHH, as in CTH. Reliability of the TEWL-3 Contextual Writing subtest was evaluated through internal consistency reliability and interrater reliability. Internal consistency, as measured by Cronbach's alpha, was .957 overall, which indicated excellent internal consistency reliability on the TEWL-3 Contextual Writing subtest. Cronbach's alpha was also excellent across all three groups: CTH = .963, CI = .921, HA = .933. Thus, the TEWL-3 Contextual Writing subtest has high internal consistency for both CTH and children who are DHH.

Cohen's weighted kappa with quadratic weights was used to determine if there was agreement between the multiple raters' scores on the TEWL-3 Contextual Writing subtest. There was a statistically significant agreement between the first author and the second author (κ = .924, 95% confidence interval [.885, .962], *p* < .001). The strength of the agreement was classified as very good. Cohen's weighted kappa was also very good across groups: CTH = .887, CI = .912, HA = .954. A third SLP, who did not complete the lab's scoring training and had a decade of experience as a school-based SLP, also scored a random 33% of each group. There was also a statistically significant agreement between the trained and untrained SLP raters (κ = .933, 95% confidence interval [.888, .979], CTH = .957, CI = .904, HA = .884).

### Research Question 2: Does the TEWL-3 Have Strong Concurrent Validity for Assessing Writing in Children Who Are DHH, as in CTH?

Validity of the TEWL-3 Contextual Writing subtest was evaluated through concurrent validity by comparing the relation between the TEWL-3 Contextual Writing subtest and one assessment measuring the same construct (written expression) and scored from the same written product as the TEWL-3; one assessment measuring a similar construct (writing fluency) and scored from a different written product; one assessment measuring a separate but related construct to writing (reading); and one assessment measuring a construct that should be minimally related to writing (math computation). First, a Pearson's correlation was run to assess the relation between the TEWL-3 Contextual Writing raw scores and the 6 + 1 Trait Writing Rubric: Grades K–2 total scores. There was a statistically significant, strong positive correlation between the raw scores from the TEWL-3 Contextual Writing subtest and the total scores from the 6 + 1 Trait Writing Rubric: Grades K–2 (Cohen, 1988). Pearson's correlations for the TEWL-3 Contextual Writing subtest and the 6 + 1 Trait Writing Rubric: Grades K–2 are outlined by group in Table 4.

**Table 4.** Pearson's correlations for the Test of Early Written Language–Third Edition (TEWL-3) Contextual Writing by group.

Assessment	TEWL-3 scores by group			
	All participants	CTH	CI	HA
6 + 1 Trait Writing Rubric: K–2	.902**	.926**	.914**	.887**
KTEA-3				
Math computation	.380**	.071	.584**	.465*

*Note.* CTH = children with typical hearing; CI = cochlear implant users; HA = hearing aid users; KTEA-3 = Kaufman Test of Educational Achievement–Third Edition.

* Statistically significant at the *p* < .05 level.

** Statistically significant at the *p* < .001 level.

Second, a Spearman's rank-order correlation was completed to determine the relation between the TEWL-3 Contextual Writing raw scores and the Writing CBM component scores. A statistically significant, moderate correlation existed between the TEWL-3 Contextual Writing raw scores and the Writing CBM component scores for all participants (Cohen, 1988). Spearman's rank-order correlations for the TEWL-3 Contextual Writing subtest and the Writing CBM components are outlined by group in Table 5.

**Table 5.** Spearman's rank-order correlations for the Test of Early Written Language–Third Edition (TEWL-3) Contextual Writing by group.

Assessment	TEWL-3 scores by group			
	All participants	CTH	CI	HA
Writing CBM components				
Total words written	.530**	.496**	.509*	.444*
Words spelled correctly	.595**	.586**	.522*	.593**
Writing sequences	.598**	.569**	.558*	.478**
WRMT-III				
Total reading	.440**	.526**	.482**	.232

*Note.* CTH = children with typical hearing; CI = cochlear implant users; HA = hearing aid users; CBM = Curriculum-Based Measure; WRMT-III = Woodcock Reading Mastery Tests–Third Edition.

* Statistically significant at the *p* < .005 level.

** Statistically significant at the *p* < .001 level.

Third, a Spearman's rank-order correlation was completed to determine the relation between the TEWL-3 Contextual Writing raw scores and the WRMT-III Total Reading index scores. A statistically significant, moderate correlation existed between TEWL-3 Contextual Writing raw scores and the WRMT-III Total Reading index scores for the CTH and CI groups. Notably, there was minimal to no correlation between TEWL-3 Contextual Writing raw scores and the WRMT-III Total Reading index scores for the HA group (Cohen, 1988). Spearman's rank-order correlations for the TEWL-3 Contextual Writing subtest and the WRMT-III Total Reading index are outlined by group in Table 5.

Last, a Pearson's correlation was completed to determine the relation between the TEWL-3 Contextual Writing raw scores and the KTEA-3 Math Computation standard scores. There was no correlation between the TEWL-3 Contextual Writing subtest raw scores and the KTEA-3 Math Computation standard scores for the CTH group. However, there was a statistically significant, moderate correlation between the TEWL-3 Contextual Writing raw scores and the KTEA-3 Math Computation standard scores for both DHH groups (Cohen, 1988). Pearson's correlations for the TEWL-3 Contextual Writing subtest and the KTEA-3 Math Computation subtest are outlined by group in Table 4.

### Research Question 3: Do the Standard Scores Derived From the TEWL-3 Accurately Identify Children Who Are DHH Who Do Not Meet Grade-Level Writing Expectations According to a Criterion-Referenced Measure and Do Those Outcomes Differ From CTH?

To address the third research question regarding whether the TEWL-3 Contextual Writing subtest standard scores accurately identify DHH children who do not meet grade-level writing expectations, we compared score descriptors from the norm-referenced TEWL-3 Contextual Writing subtest and the criterion-referenced 6 + 1 Trait Writing Rubric: Grades K–2. These results were then compared to the results from children in the CTH group. Per the TEWL-3 manual, the TEWL-3 Contextual Writing standard scores were categorized as below average (i.e., SS = 84 or below), average (i.e., SS = 85–115), and above average (i.e., SS = 116 or greater). Overall, at least 90% of participants from each group scored in the average or above average range on the TEWL-3 Contextual Writing subtest.

The same writing samples from the TEWL-3 Contextual Writing subtest were also given a categorical descriptor based on the average score derived from the 6 + 1 Trait Writing Rubric: Grades K–2. According to the rubric, a score of 1–3 was considered not proficient and a score of 4–6 was considered proficient. We modified the categorical descriptors by adding a borderline category: a score of 1–2.99 was considered not proficient, a score of 3–3.99 was considered borderline, and a score of 4–6 was considered proficient. On the 6 + 1 Trait Writing Rubric: Grades K–2, 68% of the CTH participants received a borderline or proficient score, whereas less than half of CI and HA participants yielded a borderline or proficient score. Writing sample score descriptors for the TEWL-3 Contextual Writing subtest and the 6 + 1 Trait Writing Rubric: Grades K–2 are outlined in Table 6. Refer to Supplemental Material S1 for written product examples and scoring according to both the TEWL-3 Contextual Writing subtest and the 6 + 1 Trait Writing Rubric: Grades K–2 for each example.

**Table 6.** Frequencies and percentages of second-grade Test of Early Written Language–Third Edition (TEWL-3) Contextual Writing and 6 + 1 Trait Writing Rubric: K–2 ratings by group.

Writing assessment	Group		
	CTH	CI	HA
TEWL-3 Contextual Writing ratings			
Below average	1 (2%)	2 (6%)	3 (10%)
Average	23 (46%)	12 (38%)	18 (62%)
Above average	26 (52%)	18 (56%)	8 (28%)
6 + 1 Trait Writing Rubric: K–2 rating			
Not proficient	16 (32%)	17 (53%)	18 (62%)
Borderline	26 (52%)	14 (44%)	11 (38%)
Proficient	8 (16%)	1 (3%)	0 (0%)

*Note.* CTH = children with typical hearing; CI = cochlear implant users; HA = hearing aid users.

## Discussion

The primary purpose of this study was to determine if the TEWL-3 Contextual Writing subtest is a reliable and valid writing assessment to use with children who are DHH, as in CTH. Internal consistency reliability and interrater reliability were strong across CTH, CI, and HA groups. Concurrent validity was strong for the CTH participants, but the pattern differed for the DHH participants. Additionally, the categorical descriptors on the norm-referenced TEWL-3 Contextual Writing subtest and the criterion-referenced 6 + 1 Trait Writing Rubric: Grades K–2 were markedly different. When using the same written product, children who scored within or above the average range on the TEWL-3 Contextual Writing subtest routinely scored in the not proficient range on the 6 + 1 Trait Writing Rubric: Grades K–2. This was true across all groups.

The TEWL-3 Contextual Writing subtest had excellent internal consistency reliability and very good interrater reliability across second grade CTH, CI, and HA groups. Cronbach's alpha was above .9 for all groups, indicating high internal consistency reliability. That is, the test items on the TEWL-3 consistently measured the same construct, written expression skills, for both DHH and CTH. Using the TEWL-3 Contextual Writing Expanded Scoring Guide (Hresko et al., 2012) and the additional scoring guidelines established during training to score the TEWL-3 written products, the first and second authors had very good interrater reliability across second-grade CTH, CI, and HA groups. Additionally, using only the TEWL-3 Contextual Writing Expanded Scoring Guide (Hresko et al., 2012) to score written products, a third SLP with school-based experience had very good interrater reliability with the first author across all groups.

When concurrent validity for the TEWL-3 Contextual Writing subtest was assessed with second-grade CTH and DHH participants, results for the CTH participants suggested high concurrent validity. However, for the DHH participants, correlations did not always follow expected patterns. For CTH participants, analyses of concurrent validity indicated that the TEWL-3 Contextual Writing subtest had a strong positive correlation with the 6 + 1 Trait Writing Rubric: Grades K–2, an assessment measuring the same construct (written expression). Additionally, concurrent validity analyses for CTH indicated a moderate positive correlation with the Writing CBM and the WRMT-III Total Reading index, assessments measuring separate but similar constructs (writing fluency and reading, respectively). Last, there was no correlation with the KTEA-3 Math Computation subtest, a construct measuring a minimally related construct (math computation). For the DHH participants, the correlations between the writing measures were similar to those for the CTH participants. However, correlations between the TEWL-3 Contextual Writing subtest and the WRMT-III Total Reading index were minimally related for the HA group. Additionally, correlations between the TEWL-3 Contextual Writing subtest and the KTEA-3 Math Computation subtest were moderately related for both DHH groups.

For the second-grade CTH group, correlations between the TEWL-3 Contextual Writing subtest and additional literacy and math assessments were generally what was to be expected for a target assessment to have strong concurrent validity. However, for the CI and HA groups, correlations between the TEWL-3 Contextual Writing subtest and the WRMT-III Total Reading index and the KTEA-3 Math Computation subtest did not follow the same pattern. Perhaps the difference in correlations between the CTH and DHH groups was due to the influence of language knowledge on each of these academic constructs. For the most part, CTH had a solid foundation of language and used that foundation of language to tap into academic skills. However, past research indicated that there was more variability in the language skills of children who were DHH (Lund, 2016; Wake et al., 2005; Werfel & Douglas, 2017; Werfel et al., 2022). Thus, instead of language skills being the foundation from which academic skills were developed, some children who were DHH may have had a lack of access to language, which could have impeded their development across all three measured skills (writing, reading, and math computation). Therefore, literacy skills and math skills may be more likely to be correlated across DHH than CTH given their reliance on foundational language knowledge that cannot be assumed for children who are DHH.

Most prior research indicated that children who were DHH demonstrated writing outcomes that were significantly below age-matched peers (Breland et al., 2022; Geers & Hayes, 2011; Mayer et al., 2016; Spencer et al., 2003). However, some groups reported no difference on writing outcomes for children who were DHH (Mayer & Trezek, 2023; Spencer et al., 2004; Tomblin et al., 2020). Different measurement approaches may explain these differences. Mayer and Trezek (2023), Spencer et al. (2004), and Tomblin et al. (2020) utilized a norm-referenced measure to assess the writing skills of children who were DHH; however, Breland et al. (2022), Geers and Hayes (2011), Mayer et al. (2016), and Spencer et al. (2003) used criterion-referenced measures. Furthermore, when researchers compared writing outcomes of CI users who used spoken English to an age-matched comparison group of typical hearing peers rather than relying on testing norms, children with CIs demonstrated poorer writing skills than their typical hearing counterparts (Breland et al., 2022; Geers & Hayes, 2011; Spencer et al., 2003).

Our findings support this conclusion. In the current study, second-grade children who were DHH obtained high-average standard scores (i.e., *M* = 111.4) on the TEWL-3 Contextual Writing subtest. Based on the categorical descriptors of standard scores derived from the manual for the TEWL-3 Contextual Writing subtest, the current study's results appeared to align with prior studies that measured writing outcomes with norm-referenced assessments (Mayer & Trezek, 2023; Spencer et al., 2004; Tomblin et al., 2020). However, when examining the current study's results through the categorical descriptors of the criterion-referenced 6 + 1 Trait Writing Rubric: Grades K–2, the results were more aligned with the below average writing outcomes found by Breland et al. (2022), Geers and Hayes (2011), Mayer et al. (2016), and Spencer et al. (2003).

This misalignment of categorical descriptors between the TEWL-3 Contextual Writing subtest and 6 + 1 Trait Writing Rubric: Grades K–2 is exemplified in three written products (i.e., one HA, one CI, and one CTH) in Supplemental Material S1. In the HA written story, the child simply described the picture devoid of most literary elements, lacked grammatical structure, and displayed limited mechanics and spacing. Using the TEWL-3 Contextual Writing scoring guidelines, the child received a standard score of 95, with the categorical descriptor of average. However, when the same written product was scored with the 6 + 1 Trait Writing Rubric: Grades K–2 scoring guidelines, the child received an average score of 2.23, with the categorical descriptor of not proficient. The HA story had a much lower score on the 6 + 1 Trait Writing Rubric: Grades K–2 due to that measure outlining specific criteria for transitions, vocabulary, sentence structure, rhythm, voice, handwriting, and spacing that are either not addressed or more generally addressed on the TEWL-3 Contextual Writing subtest. This discrepancy in scoring descriptors between the two writing assessments is concerning considering both measures are often used to make eligibility decisions.

Despite using the same written product, the majority of the current study's DHH scores fell within the average range according to testing norms on the TEWL-3 Contextual Writing subtest, yet the DHH groups' writing outcomes were poorer than those from the typical hearing age-matched comparison group according to the criterion-referenced 6 + 1 Trait Writing Rubric: Grades K–2. Because the 6 + 1 Trait Writing Rubric: Grades K–2 aligns with most of the language and writing CCSS (Education Northwest, 2021), performance descriptors from the 6 + 1 Trait Writing Rubric: Grades K–2 appear to be more indicative of grade-level writing expectations. Score descriptor discrepancies between the norm-referenced TEWL-3 Contextual Writing subtest and the criterion-referenced 6 + 1 Trait Writing Rubric: Grades K–2 provide evidence that norm-referenced measures may not be the best option, or an option to use in isolation, when making eligibility decisions related to the writing skills for children who are DHH.

When the TEWL-3 Contextual Writing and 6 + 1 Trait Writing Rubric: Grades K–2 score descriptors were compared, substantial differences in defined performance within each group resulted. The authors' third research question asked if the TEWL-3 Contextual Writing standard scores accurately identify DHH children who are not meeting grade-level expectations according to a criterion-referenced measure. The same written product was used to compare score descriptors from the norm-referenced TEWL-3 Contextual Writing subtest and the criterion-referenced 6 + 1 Trait Writing Rubric: Grades K–2, a commonly used writing progress monitoring tool in the schools. Of the 61 second-grade DHH writing samples, 92% yielded standard scores in the above-average (i.e., 43%) and average (i.e., 49%) ranges on the TEWL-3 Contextual Writing subtest whereas only 43% yielded scores in the proficient (i.e., 2%) and borderline (i.e., 41%) ranges on the 6 + 1 Trait Writing Rubric: Grades K–2. When dividing the DHH group into CI users and HA users, 56% of CI users and 28% of HA users yielded standard scores in the above-average range, and 38% of CI users and 62% of HA users yielded standard scores in the average range on the TEWL-3 Contextual Writing subtest. However, percentages between the CI group (i.e., proficient = 3%, borderline = 44%) and HA group (i.e., proficient = 0%, borderline = 38%) were more aligned on the 6 + 1 Trait Writing Rubric: Grades K–2. The results from the TEWL-3 Contextual Writing subtest indicated that most children within each group had above-average or average writing skills, with the CTH and CI groups having performed more similarly than the HA group. The opposite trend occurred with the 6 + 1 Trait Writing Rubric: Grades K–2 ratings; most students' writing skills were rated as borderline or not proficient, with the CI and HA groups having performed more similarly. The categorical descriptors of the two measures leads to two vastly different eligibility decisions.

Norm-referenced measures, such as the TEWL-3, compare the performance of an individual against a representative sample of their peers and they help identify low-performing children (Miller 1989/2019). However, according to the NAEP writing data, most children in the United States do not meet grade-level writing expectations (NCES, 2012; White et al., 2015). Therefore, norm-referenced writing assessments measure the performance of children against other children who do not meet grade-level writing expectations. This likely leads to a skewed distribution of standard scores wherein the range of average does not equate to mastery of specified skills. Thus, using a norm-referenced writing assessment to determine if children are meeting grade-level writing expectations or are eligible for specialized writing instruction is not recommended because norm-referenced tests do not describe skill acquisition based on curriculum-related benchmarks but, instead, examine performance relative to a comparison group who largely do not meet expectations.

Alternatively, criterion-referenced measures, such as the 6 + 1 Trait Writing Rubric: Grades K–2, examine the performance of children against predetermined benchmarks or competencies that represent a hierarchy of a child's mastery of a particular skill set (Miller, 1989/2019). Specifically, the 6 + 1 Trait Writing Rubric: Grades K–2 aligns with most of the CCSS in writing and language (Education Northwest, 2021) and incorporates components from the NAEP Writing Assessment Framework (Bridges, n.d.). Therefore, scores derived from the criterion-referenced 6 + 1 Trait Writing Rubric: Grades K–2 may align more closely with school expectations of a child's writing abilities than the norm-referenced TEWL-3 Contextual Writing subtest. Thus, we encourage SLPs to not rely on standard scores derived from norm-referenced measures when making eligibility decisions related to the writing abilities of children.

For the past 15 years, evidence has suggested that SLPs and related professionals should not exclusively use norm-referenced measures to make clinical decisions about the language skills of children (Carden et al., 2024; Ebert & Scott, 2014; Spaulding, 2012; Werfel & Douglas, 2017). Severity classifications on norm-referenced assessments may be more accurate for children who are typically developing than for those with language impairments (Spaulding, 2012). Additionally, a child's performance on norm-referenced language measures does not always align with their spontaneous language (Ebert & Scott, 2014; Werfel & Douglas, 2017). For the DHH population, the listening, language, and learning demands of a typical classroom may not be captured on a norm-referenced measure (Carden et al., 2024). Therefore, when making eligibility decisions and clinical impressions about a child's language skills, it is important to consider an assessment's sensitivity to accurately identify a student's strengths and needs and not depend on standard scores to ensure the child is receiving the most appropriate educational support.

The current study found that the norm-referenced TEWL-3 Contextual Writing subtest is reliable, yet we caution that the test manual descriptors of standard scores may lead to erroneous conclusions. The primary purpose of norm-referenced assessments is to rank an individual compared to a comparison group rather than measuring the individual's knowledge or skills relative to agreed-upon expectations for their age or grade (Miller, 1989/2019). However, the NAEP writing data reported that only one third of fourth-grade students (White et al., 2015) and one fourth of eighth- and 12th-grade students (NCES, 2012) demonstrated proficient writing skills. Thus, the norm-referenced TEWL-3 Contextual Writing standard scores were scaled on children who, overall, were not meeting proficient or grade-level writing expectations. Therefore, this interpretation of standard scores inflates writing abilities of all children and furthers the argument that norm-referenced measures should not be used in making eligibility decisions about children's writing skills. Rather, a criterion-referenced measure that outlines grade-level writing expectations is better suited to describe writing skill acquisition and determine eligibility for specialized writing intervention for children.

### Implications for Clinical Practice and Future Research

Based on the current study's findings, writing skills for the DHH population should continue to be addressed in clinical practice and in future research. When children who are DHH meet oral language or early reading goals in elementary school, they are often deemed to no longer require the services of an SLP. However, outcomes from this study add to the growing body of evidence that determined children who are DHH largely do not meet grade-level writing expectations and do not demonstrate writing outcomes commensurate with their age-matched peers. Therefore, SLPs should continually advocate to improve the written language skills of their pediatric DHH students by collaborating with the child, their guardians, and their teachers throughout their students' academic years (Schuele, 2017). When assessing writing outcomes for the pediatric DHH population, it is recommended that SLPs consider a writing measure that aligns with the CCSS or other predetermined grade-level competencies. Future research should compare outcomes on norm-referenced writing assessments to outcomes on criterion-referenced writing assessments to determine validity of results for all populations. When examining writing outcomes for the DHH population, subsequent research should divide the DHH participants into groups based on amplification type and include an age-matched control group of CTH to allow for direct comparison of results.

The current study provides evidence that criterion-referenced writing measures may offer a more pragmatic and authentic description of a child's writing abilities compared to norm-referenced writing measures. One study limitation is that although the test administrators were certified SLPs who went through extensive training, fidelity of administration was not monitored.

## Conclusions

Most prior research has reported that children who were DHH perform below average on writing measures. The TEWL-3 is a commonly used norm-referenced writing assessment that measures narrative discourse abilities through story writing. The TEWL-3 Contextual Writing subtest showed excellent internal consistency reliability and very good interrater reliability across CTH, CI, and HA groups. For the CTH population, correlations between the TEWL-3 Contextual Writing subtest and additional literacy and math measures indicated that the TEWL-3 Contextual Writing subtest had strong concurrent validity. However, correlations varied for the CI and HA groups. Categorical descriptors of the TEWL-3 Contextual Writing standard scores found that over 90% of second-grade children who were DHH demonstrated above-average or average writing outcomes, with no significant group differences between CTH and CI users. However, when the same written products were scored using the criterion-referenced 6 + 1 Trait Writing Rubric: Grades K–2, only 43% of second-grade DHH children demonstrated proficient or borderline writing skills, with no significant group differences between the CI users and HA users. Thus, even though norm-referenced measures may have strong reliability and validity, given the concerns raised by these findings' score descriptors and considering the purpose of norm-referenced tests, SLPs should exercise caution when using norm-referenced standardized scores to make eligibility decisions related to specialized writing intervention for all children, especially the DHH population.

## Data Availability Statement

The data sets generated and/or analyzed during the current study are available from the corresponding author upon reasonable request.

## Supplementary Material

10.1044/2025_LSHSS-25-00009SMS1 Supplemental Material S1 Examples of TEWL-3 Contextual Writing and 6+1 Trait Writing Rubric: K-2 scoring for participants' written products.
