# Supplementary material for: The Insufficiency of Norm-Referenced Writing Assessment for Identifying Writing Weaknesses in Children Who Are Deaf and Hard of Hearing
Source: Lang Speech Hear Serv Sch. 2025 Jul 25;56(4):1054–68. doi: 10.1044/2025_LSHSS-25-00009 (PMC12533574; doi:10.1044/2025_LSHSS-25-00009)
Supplement: Supplemental Material S1 [file LSHSS-56-1054-s001.pdf]

Supplemental Material S1. Examples of TEWL-3 Contextual Writing and 6+1 Trait Writing Rubric: K-2 scoring for participants' written products.

Grade 2 HA Participant
TEWL-3 Contextual Writing Standard Score: 95, average
6+1 Trait Writing Rubric: K-2 Score: 2.23, not proficient

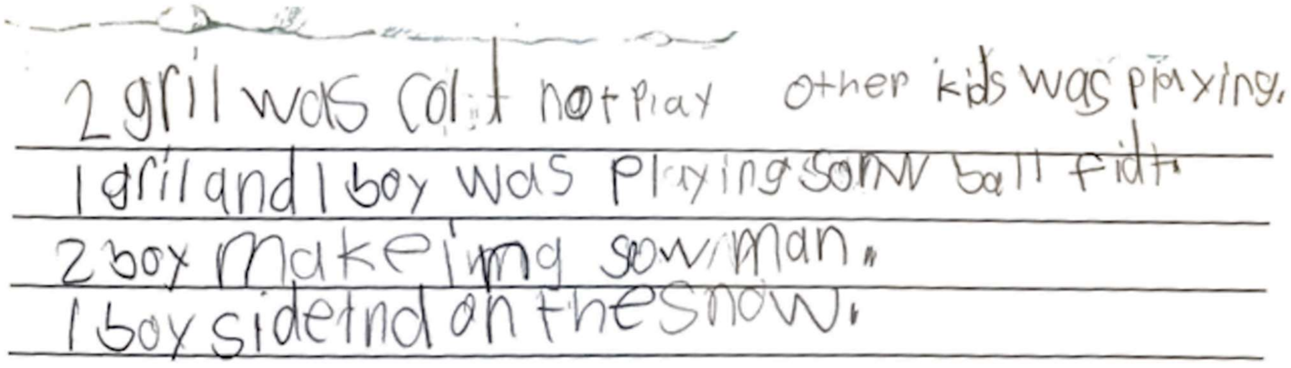

Grade 2 CI Participant
TEWL-3 Contextual Writing Standard Score: 124, above average
6+1 Trait Writing Rubric: K-2 Score: 3.23, borderline

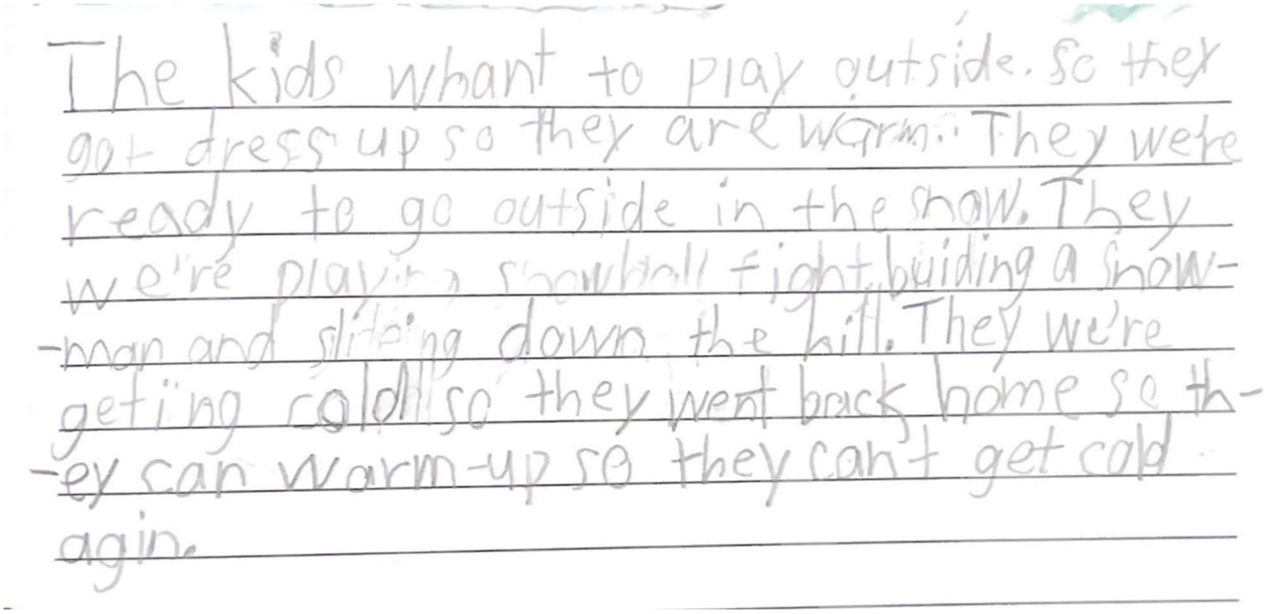

Grade 2 CTH Participant

TEWL-3 Contextual Writing Standard Score: 111, average
6+1 Trait Writing Rubric: K-2 Score: 2.86, not proficient

joy looks cool and so does
anan jock and eva arek haveing
a fun day and pip is slideing
thay all LOOK happy and taloyer
und whit are makeing a snow
man, and there is a lott of
snow that is falling from
the ski. And the chilen are at
a bus stop with owt a perent
and there are no leafs
on the tree branches.
